# Cardiovascular magnetic resonance and PET-CT of left atrial paraganglioma

**DOI:** 10.1186/1532-429X-12-1

**Published:** 2010-01-04

**Authors:** Anderanik Tomasian, Chi Lai, Stefan Ruehm, Mayil S Krishnam

**Affiliations:** 1 Department of Radiological Sciences, University of California at Los Angeles, USA; 2 Department of Pathology, University of California at Los Angeles, USA

## Abstract

Cardiac paragangliomas are among the rarest primary cardiac tumors. We present a case of left atrial paraganglioma in a patient who presented with symptoms and signs of catecholamine excess in which cardiovascular magnetic resonance in multiple orientations and PET-CT played an important role in the diagnosis and tissue characterization.

## Introduction

Pheochromocytomas or functioning paragangliomas are catecholamine-producing tumors arising from secretory chromograffin cells of neuroectodermal origin. These lesions commonly originate from the adrenal medulla. Approximately 18% of pheochromocytomas are extra-adrenal [1].Primary cardiac paragangliomas are extremely rare with less than 50 cases reported in the literature [2]. Recent advances in cross sectional imaging have provided the potential for non-invasive and accurate diagnosis of these lesions [3-5]. In this report, we describe a left atrial paraganglioma, characterized in a multi-modality setting and confirmed on histopathology.

## Case report

**Figure 1:**
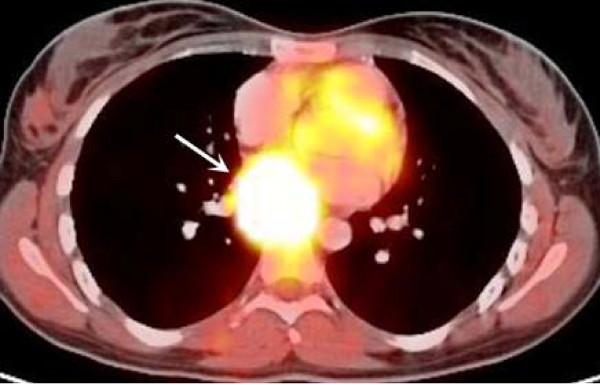
**Axial view of PET-CT scan image demonstrates a large hypermetabolic mass with intense uptake of F^18 ^Levo-DOPA in the posterior mediastinum near the left atrium which is consistent with paraganglioma**.

A 25-year-old female in her third trimester of pregnancy, presented with paroxysmal headache, palpitations, sweating, and hypertension of up to 230/130 for three weeks. Her medical history was negative for previous hypertension, and physical examination was unremarkable. Initial work-up for hypertension excluded pre-eclampsia. Further biochemistry evaluation revealed markedly elevated urine catecholamines;

Dopamine 607 μg/24 hours (range 65 - 400), Norepinephrine 978 μg/24 hours (range 15 - 80), Metanephrine 108 μg/24 hours (range 24 - 96), Normetanephrine 4067 μg/24 hours (range 75 - 375), and Vanillyl mandelic acid 13.6 mg/24 hours (range 2 - 7). Epinephrine level was 0.8 μg/24 hours (range 0.0-20.0). A diagnosis of pheochromocytoma was considered and the patient underwent abdominal magnetic resonance imaging (MRI) which demonstrated a 28 × 19 mm left juxta-adrenal mass. Treatment with phenoxybenzamine (10 mg, twice a day) was initiated. Six weeks following uneventful delivery by Cesarean Section, the patient underwent left laparoscopic adrenalectomy for suspected pheochromocytoma. Histopathology showed normal adrenal gland and brown fat in the juxta-adrenal mass. The patient's symptoms continued, and with the suspicion of extra-adrenal pheochromocytoma, F^18 ^Levo-DOPA positron emission tomography-computed tomography or PET-CT scan of the whole body was performed to localize the lesion (Siemens Medical Solutions, Erlangen, Germany). The intravenous F^18 ^Levo-DOPA dose was 6.9 mCi, and the patient was pre-medicated with 200 mg of carbidopa 45 minutes before the scan to enhance the uptake of F^18 ^Levo-DOPA. On PET-CT, a large hyper-metabolic soft tissue mass measuring 4.5 × 2.7 × 3.9 cm in the region of posterior mediastinum near the left atrium was noted (Figure 1, arrow).

**Figure 2:**
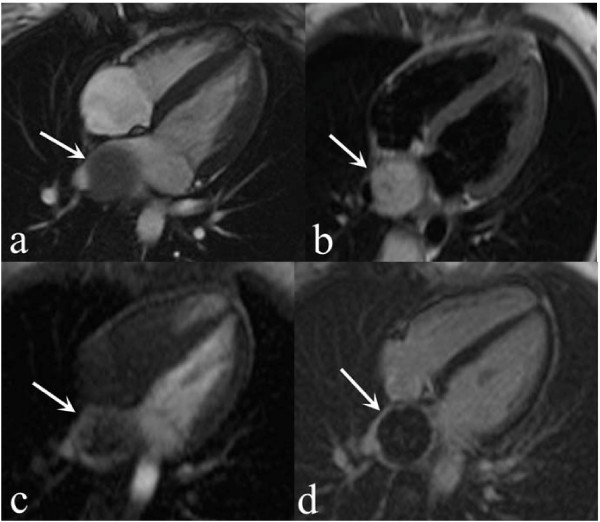
**Horizontal long axis CMR images demonstrate a round, well defined posterior mediastinal mass with smooth extrinsic compression of left atrial wall which is hypo-intense on breath-hold Steady State Free Precession (SSFP) cine CMR (a, arrow), and shows homogenously high signal on T2-weighted dark blood turbo spin-echo image (b, arrow)**. T1-weighted inversion recovery gradient-echo myocardial perfusion imaging demonstrates heterogeneous, mostly peripheral, dynamic contrast filling of the mass suggesting vascularity (c, arrow), and T1-weighted, fat-saturated, inversion recovery late gadolinium enhancement image shows peripheral rim enhancement with no evidence of central enhancement suggestive of central tissue necrosis (d, arrow).

Four days following the PET-CT examination, cardiovascular MR (CMR) was performed on a 1.5 Tesla MR scanner (Magnetom Avanto, Siemens Medical Solutions, Malvern, Pennsylvania, USA) to further characterize the lesion and its anatomical relation to the atria and surrounding structures. On Steady State Free Precession (SSFP) breath-hold cine MRI (TE: 1.2 ms, TR: 2.4 ms, temporal resolution: 40.3 ms), the epicenter of the hypo-intense soft tissue mass was noted to be in the posterior mediastinum with no apparent fat plane between the lesion and the posterior wall of the left atrium (Figure 2-a, arrow). The mass caused smooth extrinsic compression of left atrial wall and demonstrated typical low signal on T1-weighted spin-echo (TE: 14 ms, TR: 750 ms) and high signal on T2-weighted dark blood turbo spin-echo (TE: 86 ms, TR: 4000 ms) images (Figure 2-b).

**Figure 3:**
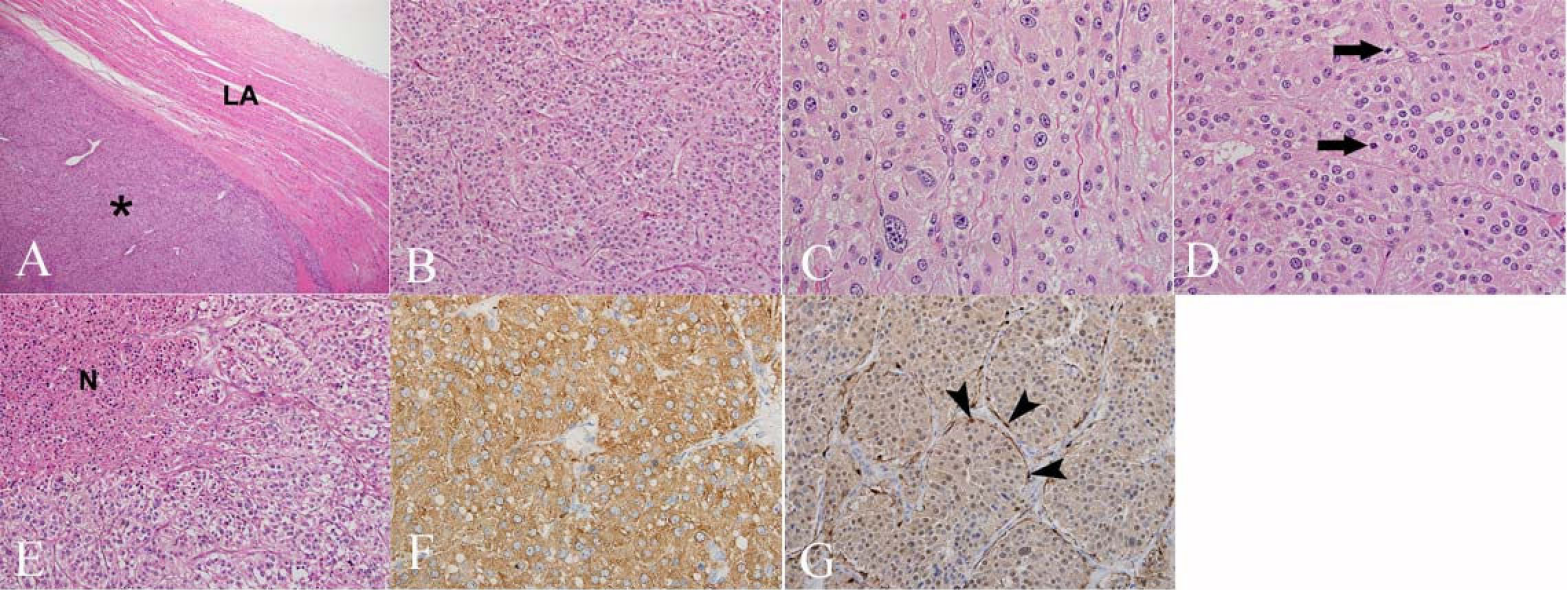
(A) Low power photomicrograph (40×; H&E stain) demonstrates tumor (*) compressing left atrium (LA). **(B) **The tumor is comprised of nests of fairly uniform cells with moderate eosinophilic, granular cytoplasm (200×; H&E stain). **(C) **Marked cytologic atypia with pleomorphic nuclei and prominent nucleoli was focally present (400×; H&E stain) in addition to **(D) **increased number of mitoses (arrows) (400×; H&E stain). **(E) **Scattered foci of coagulative tumor necrosis (N) were also noted (200×; H&E stain). **(F) **The tumor exhibited diffuse, strong cytoplasmic positivity for synaptophysin immunostain (400×). **(G) **S100 immunostain highlights sustentacular cells surrounding nests of tumor cells (400×).

During administration of 5 mL of gadopentetate dimeglumine (Magnevist, Berlex Laboratories, Wayne, NJ), T1-weighted inversion recovery gradient-echo myocardial perfusion imaging was performed which demonstrated heterogeneous dynamic contrast filling of the mass (Figure 2-c). In addition, 10 minutes following injection of 0.2 mmol/kg body weight of Magnevist, late gadolinium enhancement (LGE) CMR was performed using T1-weighted, fat-saturated, inversion recovery sequence. This demonstrated peripheral rim enhancement of the mass with central dark signal suggestive of central tissue necrosis (Figure 2-d). Based on clinical history, urine biochemistry, and radiological features, a diagnosis of cardiac paraganglioma, although very rare, was considered. Surgical procedure was performed through a right anterolateral thoracotomy, and following cardio-pulmonary bypass, the tumor was excised. At surgery, the left atrial mural mass was 4.5 cm in largest dimension originating in the posterior wall with extension to Sondergaard's groove and inter-atrial septum. Following excision, left atrial wall and pericardial reconstructions were performed using bovine pericardium and Gore-Tex patch, respectively. Two days after surgery, 24-hour urine catecholamines returned to normal range, and following uneventful recovery, the patient was discharged in stable condition. Histopathologic evaluation indeed confirmed a cardiac paraganglioma (Figure 3).

The patient remained asymptomatic during the 4-month follow-up period without local recurrence on echocardiography.

## Discussion

Primary cardiac tumors are rare with a reported prevalence of 0.001% to 0.03% on autopsy series [6]. Of these tumors, cardiac paragangliomas are among the rarest accounting for less than 1% of cases [6]. These tumors originate from paraganglial cells of the arteries or the visceral autonomic paraganglia of the atria [7]. Cardiac paragangliomas are most commonly located in the left atrium [1] and less frequent sites include right atrium, inter-atrial septum, and the left ventricle [1,4,8]. The mean age at the presentation is 36-40 years with approximately equal sex distribution [9]. Cardiac paragangliomas are catecholeamine-secreting in 35-50% of cases [6].

Clinical presentations of cardiac paraganglioma range from an incidental finding [9] to constitutional symptoms including malaise, weight loss and fever [3], symptoms and signs of catecholamine excess (paroxysmal headache, palpitation, sweating, and hypertension) [1], arrhythmia due to invasion of the conductive system [4], angina pectoris or myocardial infarction secondary to compression or obstruction of coronary arteries [5], acute heart failure or dilated cardiomyopathy [10], pericardial involvement, and disruption of valvular function [4].

The incidence of malignancy is reported to be approximately 10% [6]. However, there are no histological criteria to differentiate benign from malignant cardiac paragangliomas [11]. Malignancy is determined by tumor behavior rather than histological appearance and the distinguishing feature includes presence of distant metastasis [11]. Histopathologically, paragangliomas are monomorphous tumors composed of nests of paraganglial cells (Zellballen) surrounded by sustentacular cells [12].

Cardiac paragangliomas are echogenic masses on echocardiography and are reported as circumscribed, heterogeneous masses with low attenuation on un-enhanced CT [14] with marked enhancement on contrast enhanced CT [12].

These lesions can be localized using very specific radiotracers such as ^131^I or ^123^I metaiodobenzylguanidine or ^18^F-DOPA, which are actively transported into neurosecretory granules of catecholamine-producing cells [13]. ^18^F-DOPA PET studies have yielded promising results in the imaging of pheochromocytoma [13], demonstrating typical intense uptake of ^18^F-DOPA by the paraganglional cells of the lesions, as showed in our case.

Due to its higher spatial resolution and multiplanar image acquisition, CMR is considered an important technique in diagnosis and characterization of cardiac tumors. Cine SSFP and pre-contrast T1- and T2-weighted imaging are useful for evaluation of anatomic features, whereas first pass dynamic perfusion and late gadolinium enhancement sequences are important in assessment of vascularity.

As seen in our patient, cardiac paragangliomas are typically iso- or hypo-intense relative to myocardium on T1-weighted, and markedly hyper-intense on T2-weighted images [14]. They are highly vascular and as in our case, may typically demonstrate dynamic contrast filling on myocardial perfusion imaging which is often heterogeneous, with central non-enhancing areas suggesting tumor necrosis [14]. Presence of peripheral rim enhancement on LGE imaging also indicates vascularity of these lesions distinguishing them from avascular cardiac masses such as thrombi and lipoma. Although presence of central dark signal on LGE could be due to nulling of signal following Inversion pulse, it likely represents tissue necrosis. Cardiac hemangiomas demonstrate LGE, intermediate and high signal intensity on T1 and T2-weighted images, respectively, and show enhancement on perfusion imaging [12]. Cardiac lymphangiomas demonstrate high signal intensity on T1 and T2 weighted images, whereas rhabdomyomas are isointense on T1 and hyper-intense on T2 weighted images compared to adjacent myocardium [14].

Surgical resection of cardiac paraganglioma results in complete cure and relief of symptoms [9,12]. Surgical risks particular to these lesions include fatal hemorrhage due to high vascularity, and hypertensive crisis from intra-operative manipulation which may be avoided by cardiopulmonary bypass allowing safe dissection. Since paragangliomas may be infiltrative lesions, as in our patient, extensive resection of the atrial wall may be required for complete excision [7].

## Conclusion

The combination of CMR with various image acquisition sequences in multiple orientations and PET-CT studies, provides a powerful tool for non-invasive morphological assessment and tissue characterization of cardiac paraganglioma, especially when correlated with clinical history and appropriate biochemistry evaluations.

## Consent

Written informed consent was obtained from the patient for publication of this case report and any accompanying images. A copy of the written consent is available for review by the Editor-in-Chief of this journal.

## Competing interests

The authors declare that they have no competing interests.

## Authors' contributions

All authors were involved in the conception and design of the manuscript, acquisition of data, drafting the manuscript, critical revision of the manuscript for intellectual content, and final approval.
